# Combination of anti-C1qA08 and anti-mCRP a.a.35-47 antibodies is associated with renal prognosis of patients with lupus nephritis

**DOI:** 10.3389/fimmu.2023.1181561

**Published:** 2023-04-17

**Authors:** Xiao-Ling Liu, Ying Tan, Feng Yu, Shang-Rong Ji, Ming-Hui Zhao

**Affiliations:** ^1^ Ministry of Education (MOE) Key Laboratory of Cell Activities and Stress Adaptations, School of Life Sciences, Lanzhou University, Lanzhou, China; ^2^ Renal Division, Peking University First Hospital, Beijing, China; ^3^ Institute of Nephrology, Peking University, Beijing, China; ^4^ Key Laboratory of Renal Disease, Ministry of Health of China, Beijing, China; ^5^ Key Laboratory of Chronic Kidney Disease (CKD) Prevention and Treatment, Ministry of Education of China, Beijing, China; ^6^ Research Units of Diagnosis and Treatment of lmmune-Mediated Kidney Diseases, Chinese Academy of Medical Sciences, Beijing, China; ^7^ Department of Nephrology, Peking University International Hospital, Beijing, China

**Keywords:** anti-C1qA08 autoantibody, anti-mCRP a.a.35-47 autoantibody, autoantibodies, renal prognosis, lupus nephritis

## Abstract

**Objective:**

The aim of this study is to explore the prevalence and clinicopathological associations between anti-C1qA08 antibodies and anti-monomeric CRP (mCRP) a.a.35-47 antibodies and to explore the interaction between C1q and mCRP.

**Methods:**

Ninety patients with biopsy-proven lupus nephritis were included from a Chinese cohort. Plasma samples collected on the day of renal biopsy were tested for anti-C1qA08 antibodies and anti-mCRP a.a.35-47 antibodies. The associations between these two autoantibodies and clinicopathologic features and long-term prognosis were analyzed. The interaction between C1q and mCRP was further investigated by ELISA, and the key linear epitopes of the combination of cholesterol binding sequence (CBS; a.a.35-47) and C1qA08 were tested by competitive inhibition assays. The surface plasmon resonance (SPR) was used to further verify the results.

**Results:**

The prevalence of anti-C1qA08 antibodies and anti-mCRP a.a.35-47 antibodies were 50/90 (61.1%) and 45/90 (50.0%), respectively. Levels of anti-C1qA08 antibodies and anti-mCRP a.a.35-47 antibodies were negatively correlated with serum C3 concentrations ((0.5(0.22-1.19) g/L vs. 0.39(0.15-1.38) g/L, *P*=0.002) and (0.48(0.44-0.88) g/L vs. 0.41(0.15-1.38) g/L, *P*=0.028), respectively. Levels of anti-C1qA08 antibodies were correlated with the score of fibrous crescents and tubular atrophy (r=-0.256, *P*=0.014 and r=-0.25, *P*=0.016, respectively). The patients with double positive antibodies showed worse renal prognosis than that of the double negative group (HR 0.899 (95% CI: 0.739-1.059), *P*=0.0336). The binding of mCRP to C1q was confirmed by ELISA. The key linear epitopes of the combination were a.a.35-47 and C1qA08, which were confirmed by competitive inhibition experiments and SPR.

**Conclusion:**

The combination of anti-C1qA08 and anti-mCRP a.a.35-47 autoantibodies could predict a poor renal outcome. The key linear epitopes of the combination of C1q and mCRP were C1qA08 and a.a.35-47. A08 was an important epitope for the classical pathway complement activation and a.a.35-47 could inhibit this process.

## Introduction

Systemic lupus erythematosus (SLE) is a systemic autoimmune disease with multiple autoantibodies. Lupus nephritis (LN) is the most prevalent secondary glomerulonephritis in China. LN is the one of the principal causes of morbidity and mortality among various major organ manifestations of lupus (1). The development of glomerulonephritis in SLE was associated with the presence of some specific nephritogenic autoantibodies, such as anti-double-stranded DNA (anti-dsDNA) antibodies (2–4), anti-Sm antibodies, anti-C1q antibodies (5–8), and anti-C-reactive protein (CRP) antibodies (9, 10), whereas more than 150 autoantibodies were reported in SLE. It is still controversial that which autoantibodies are associated with renal clinical and pathological activity and the renal outcome. At present, the exact immunological pathogenesis of SLE and LN is still unclear. It may be caused by apoptotic cell clearance encountering an immune complex in the kidney, which in turn causes complement activation to result in kidney damage.

C1q is the classical pathway promoter of complement activation and is the largest complement protein. C1q is composed of six subunits, each of which consists of three chains A, B, and C, and is divided into three parts: a head region, a collagen region, and a tail portion. C1q is the most positively charged protein in serum, and its ligands are diverse (11). The head region of C1 can be combined with antigen-antibody complex, apoptotic cells, CRP, and pentraxin-3 (PTX3), and the collagen region can be combined with C1r, C1s, and mCRP. The main physiological function of C1q is to clear immune complexes and apoptotic bodies and to exert different biological functions in combination with different ligands (12–15). Vanhecke D et al. confirmed that the linear epitope A08 located on the A chain is an important antigen recognition site for anti-C1q autoantibodies (16). Long-term follow-up studies using large cohorts in some studies have shown that anti-C1qA08 antibodies were associated with disease activity and prognosis in Chinese patients with LN (17). H. Jiang et al. demonstrated for the first time that CRP binds to C1q and that CRP mainly binds to peptides 14-26 of the C1q A-chain collagen region, which is basically identical to the amino acid sequence of the linear epitope of A08 mentioned above (18).

CRP has two opposite structural faces, the ligand binding surface, and the effective surface. When CRP binds to a ligand, its effect surface can bind to complement C1q, thereby activating the classical pathway (19). The pentameric CRP, which is expressed during body infection and systemic inflammation, is involved in the process of apoptotic cell clearance (12). With the dissociation of the pentameric structure, the conformation of the CRP subunit changes and exhibits an mCRP epitope (20). Studies have reported that damaged cell membranes can induce CRP dissociation (21). In the inflammatory microenvironment, mCRP is likely to exert pro-inflammatory effects in a “functional state”. As an acute-phase plasma protein, CRP can rapidly increase its concentration by 1000 times in inflammation (22, 23). SLE is a classic immunoinflammatory disease, but the concentration of CRP as a marker of inflammation is only slightly elevated or maintains normal levels during disease activity in SLE patients (24, 25). A previous report found that anti-mCRP antibodies were not only associated with disease activity but also with renal prognosis in LN (26), and a.a.35-47 seemed to be the most important epitope of mCRP (27).

This study investigated the associations of clinical, laboratory and pathological features and prognosis of LN with a panel of autoantibodies, including anti-C1qA08 and anti-mCRP a.a.35-47 antibodies in a large cohort of Chinese patients with LN. The combination of anti-C1qA08 and anti-mCRP a.a.35-47 antibodies could indicate higher renal disease activity and predict renal outcome. Studies on the interactions between C1q and mCRP were further explored to show their role in the development of LN.

## Material and methods

### Patients

Between January 2000 and July 2010, 90 patients with LN were diagnosed by renal biopsy and pathological examination at the First Hospital of Peking University and had complete clinical pathology and follow-up data. All patients were fulfilled the 1997 American College of Rheumatology (ACR) revised criteria for SLE (28). The SLEDAI score was used to evaluate the patient’s systemic disease activity (29, 30).

Informed consent was obtained from all the patients. The research was in compliance with the Declaration of Helsinki. The design of this work was approved by the local ethical committees. The composite endpoints were defined as death, end-stage renal disease (ESRD), ≥30% reduction from baseline estimated glomerular filtration rate (eGFR) or LN flares.

The serum of the patient was obtained on the day of the renal biopsy prior to the initiation of immunosuppressive therapy. At the same time, 60 healthy blood donors matched with the age and sex of the patients were selected as normal controls. Serum was stored in a refrigerator at -80°C after being packed to avoid repeated freezing and thawing. Kidney biopsy specimens were examined by immunofluorescence and electron microscopy. Pathological parameters, including activity indices (AI) and chronicity indices (CI), were determined by renal pathologists (31, 32).

The research was in compliance with the Declaration of Helsinki. It was approved by the ethics committee of Peking University First Hospital (No. 20161163).

### Peptides synthesis

Biotinylated and nonbiotinylated peptides (>95% purity) were synthesized by GenScript. Peptides A08 (GRPGRRGRPGLKG) and B78 (PGKVGPKGPMGPK) were derived from the collagen like region (CLR) sequences of the C1q-A chain and the C1q-B chain, respectively. Peptide A08-C (GAPGKDGYDGLPG)was derived from the C1q-C chain and located at the N-terminal region homologous to peptide A08. It was used as a negative control peptide. Peptides 35-47 (VCLHFYTELSSTR) and 199-206 (FTKPQLWP), which were derived from the mCRP. The purified peptides were then confirmed by high-performance liquid chromatography for purity and by mass spectrometry to verify the correct sequence.

### Detection of anti-C1qA08 and anti-mCRP a.a.35-47 antibodies by ELISA

The final concentration of avidin was 5μg/ml in a 96-well microtiter plate, and overnight at 4°C, and the avidin-free well was used as a non-antigen control. It was washed with PBST and then blocked with 0.1% collagen at 37°C for 1 hour. The biotin-labelled peptide A08/a.a.35-47 to 5 μg/ml was diluted with PBS, added to the plate, and incubated at 37°C for 2 hours. Avidin specifically binds to biotin to immobilize the peptide on the plate. The plasma sample (1:200) was added and diluted and incubated at 37°C for 1 hour. Alkaline phosphatase (AP)-labelled goat anti-human IgG (1:5000) was added and incubated for 1 hour at 37°C. Finally, after an alkaline phosphatase substrate solution added, the absorbance at 405 nm was measured with a microplate reader. The cut-off value was set as the mean +2SD of the 60 healthy blood donors.

### C1q binding assays

The final concentration of C1q was diluted 5 μg/ml with carbonate buffer, and 100 μl of the coated 96-well microtiter plate was taken and incubated overnight at 4°C. Wells were washed with Phosphate Buffered Solution containing 0.1% TWEEN-20 (PBST) and blocked with 1% BSA/PBS for 1 hour. Different concentrations of mCRP were added at 37°C for 1 hour. In the competition assay, mCRP was incubated with different concentrations of the relevant peptides a.a.35-47 and a.a.199-206 at 37°C for 30 minutes in each well. Binding was detected with mCRP antibody and alkaline phosphatase (AP)-labelled goat anti-mouse IgG antibody (1:5000). The absorbance at 405 nm was measured. At the same time, mCRP 4 μg/ml was diluted with carbonate buffer, fixed in a 96-well microtiter plate, and incubated overnight at 4°C. Wells were washed with PBST containing 0.1% TWEEN-20 and blocked with 1% BSA/PBS at 37°C for 1 hour, and C1q was incubated with different concentrations of related peptides A08, B78, and A08-C for 37 minutes at 37°C to each well. Binding was detected using a C1q antibody and an alkaline phosphatase (AP)-labelled goat anti-rabbit IgG antibody (1:10000), and the absorbance at 405 nm was measured.

The interaction of C1q with mCRP and peptides a.a.35-47, a.a.199-206 was detected using the Biacore T200 direct assay. Next, the interaction of mCRP with C1q and peptides A08, B78, and A08-C were examined. CRP was immobilized on a CM5 chip, followed by a 200 s denaturing agent (8 M urea and 5 mM EDTA) at a flow rate of 30 μl/min to denature CRP to mCRP, then at a flow rate of 30 μl/min in a 0.05% P-20 The analyte was injected into the PBS and only the injection buffer was used as a negative control in the other channel.

### Anti-C1qA08 antibody inhibited the binding of C1q to mCRP

The human complement component C1q (4 μg/ml) was first diluted with carbonate buffer and was coated on the wells of polystyrene microtiter plates. After blocking with 0.1% collagen at 37°C for 1 hour, each well was washed with PBS containing 0.1% TWEEN-20 (PBST). The mCRP (2 μg/ml) was added with different concentrations of C1qA08 mAb (17-9), preincubated for 30 min at 37°C, and then added to a 96-well microtiter plate. After washing, A08-specific antibody 3H12 (1:200) was diluted and added to the plate for 1 hour at 37°C. Alkaline phosphatase (AP)-labelled goat anti-mouse IgG was added and incubated for 1 hour at 37°C. The P-nitrophenyl phosphate (1 mg/ml; Sigma-Aldrich) was diluted in substrate buffer (1.0 M diethanolamine and 0.5 mM MgCl2 (pH 9.8). Then optical density was measured at 405 nm.

### C3 deposition assay

C3 deposition assay was performed as previously described by Roumenina LT et al. (33). The recombinant α3(IV)NC1 (2 μg/ml) was first diluted with carbonate buffer and coated on the wells of polystyrene microtiter plates. After blocking with 0.1% collagen at 37°C for 1 hour, each well was washed with PBS containing 0.1% TWEEN-20 (PBST). Total IgG from patients with anti-glomerular basement membrane disease and positive anti-α3(IV)NC1 autoantibody were purified by a protein G column (34) and were diluted to 20 μg/ml in PBST for the binding of anti-α3(IV)NC1 autoantibody. The plate was washed with veronal buffered saline containing 0.1% TWEEN-20 (VBST), and then normal human serum was diluted 1:100 with VBST. The diluted serum was added with different concentrations of mCRP and a.a.35-47, preincubated for 30 min at 37°C, and then added to a 96-well microtiter plate. Incubate for 1 hour at 37°C. After washing, rabbit anti-human C3c antibody (1:10000) was diluted and added to the plate for 1 hour at 37°C. Alkaline phosphatase (AP)-labelled goat anti-rabbit IgG was added and incubated for 1 hour at 37°C. The P-nitrophenyl phosphate was used in the substrate buffer. Optical density was measured at 405 nm.

### Statistical analysis

The data were analysed using SPSS 21.0 statistical software (SPSS, Inc, Chicago, IL, USA). Quantitative data were expressed as mean ± SD or median with a range minimum–maximum. For comparison of clinical features and pathologic data of patients, the 1-way analysis of variance. Spearman correlation was performed to analyse correlation. Kaplan-Meier curves were used to analyse prognosis. Univariate survival analysis was carried out with the log-rank test. Results were expressed as HR with a 95% CI. Statistical significance was considered *P* < 0.05.

## Results

### General patient data

Clinical data of 90 LN patients from the Peking University First Hospital were shown in **Table 1**. There were 15 (16.7%) male and 75 (83.3%) female patients with a median age of 29 (13 to 67) years. 20 patients were classified as class III (22.2%, including 8 as class III+V) and 70 as class IV (77.8%, including 16 as class IV +V). The demographic and clinical data were summarized in **Table 1**.

**Table 1:** General clinical profiles of patients with lupus nephritis at renal biopsy.

Clinical Evaluation		Laboratory Assessment	
Number of patients	90	Leukocytopenia, no. (%)	20(22.2)
Age (median and range) (years)	32.51(14-67)	Thrombocytopenia, no. (%)	12(13.3)
Female, no. (%)	84.4%	Hematuria, no. (%)	72(80.0)
Hypertension (BP≥140/90mmHg), no. (%)	47(52.2)	Leukocyturia, no. (%)	49(54.4)
Nephrotic syndrome, no. (%)	49(54.4)	Hemoglobin, mean ± s.d.(g/L)	108.4 ± 24.6
Acute kidney injury, no. (%)	18(20.0)	Urinary protein, median (range) (g/24h)	3.8(0.2-22.45)
Anemia, no. (%)	41(45.6)	Serum creatinine, median (range) (umol/L)	75.4(26.1-792.0)
Neurological disorder, no. (%)	5(5.6)	C3 level, median (range) (g/L)	0.5(0.15-1.38)
SLEDAI, mean ± s.d	17.4 ± 6.3	C4 level, median (range) (g/L)	0.11(0.00-1.01)
Follow-up time, m, median (range)	56.73(27-86)	ANA (+), no. (%)	87(96.7)
Duration from SLE onset to renal biopsy, (months), mean ± s.d	24.0 ± 3.9	Anti-dsDNA antibodies, no. (%)	69(76.7)

### The associations between anti-C1qA08 and anti-mCRP a.a. 35-47 autoantibodies and clinicopathologic features of LN patients

**Figure 1:**
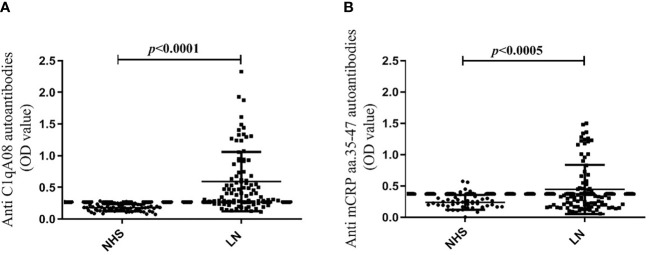
**(A)** Comparisons of levels of anti-A08 antibodies in LN and healthy blood donors. **(B)** Comparisons of levels of anti-mCRP a.a.35-47 antibodies in LN and healthy blood donors.

The cut-off values of the anti-C1qA08 antibodies and anti-mCRP a.a.35-47 antibodies were illustrated in **Figure 1**. In the discovery cohort, the anti-C1qA08 autoantibodies were detected in 55 of 90 (61.1%) patients, which was significantly higher than that in the normal healthy subjects (0/60, 0%, *P* < 0.0001). Anti-mCRP a.a.35-47 antibodies were detected in 45 of 90 (50.0%) patients, which were significantly higher than that in the normal healthy subjects (4/60, 6.67%, *P* < 0.001).

The associations between with anti-C1qA08 antibodies and anti-mCRP a.a.35-47 antibodies and clinicopathologic features were shown in **Tables 2**; **3**. Levels of anti-C1qA08 antibodies and anti-mCRP a.a.35-47 antibodies were negatively correlated with serum C3 concentrations ((0.5(0.22-1.19) g/L vs. 0.39(0.15-1.38) g/L, *P*=0.002) and (0.48(0.44-0.88) g/L vs. 0.41(0.15-1.38) g/L, *P*=0.028)). Levels of anti-C1qA08 antibodies were correlated with the score of fibrous crescents and tubular atrophy (r=-0.256, *P*=0.014 and r=-0.25, *P*=0.016, respectively).

**Table 2:** Associations of anti-C1qA08 antibodies and anti-mCRP a.a.35-47 antibodies with clinical parameters.

	Anti-mCRP a.a.35-47 -/+ (*P* value)	Anti-C1qA08-/+ (*P* value)
SLEDAI scores	16.89 ± 5.871/18.36 ± 7.127(0.283)	18.60 ± 6.273/17.18 ± 6.679(0.331)
Acute kidney injury(-/+)	10(22.2%)/9(19.1%)(0.716)	6(20%)/13(21%)(0.914)
Hemoglobin(g/L)	110(47-177)/109(60-144)(0.656)	110(63-156)/109(47-177)(0.714)
Hematuria(-/+)	35(77.8%)/39(83%)(0.530)	25(83.3%)/49(79.0%)(0.626)
Leukocyturia(-/+)	24(53.3%)/25(53.2%)(0.585)	18(60%)/31(50.0%)(0.199)
Proteinuria(g/d)	4.43(0.48-22.45)/3.5(0.23-20.62)(0.182)	4.46(0.48-15.38)/3.57(0.23-22.45)(0.157)
Serum creatinine(µmol/L)	75.7(36.7-618)/77(26.1-792)(0.984)	76.35(36.7-618)/76.4(26.1-792)(0.825)
Serum C3(g/L)	0.5(0.22-1.19)/0.39(0.15-1.38)(0.002)	0.48(0.44-0.88)/0.41(0.15-1.38)(0.028)

**Table 3:** Associations of anti-C1qA08 antibodies and anti-mCRP a.a.35-47 antibodies with renal pathology scores.

Renal pathology score	Anti-mCRP a.a.35-47 antibodies		Anti-C1qA08 antibodies	
	*r* value	*p* value	*r* value	*P* value
Activity indices score	-0.082	0.514	0.042	0.737
Cellular fiber crescent	-0.136	0.196	-0.066	0.533
Neutrophil infiltration and/or nuclear fragmentation	0.000	0.997	0.088	0.406
Wire loop/transparent thrombus	0.076	0.471	0.065	0.538
Interstitial inflammatory	-0.017	0.872	-0.144	0.172
Chronicity indices score	-0.125	0.321	-0.072	0.568
Spherical sclerosis	0.036	0.732	0.037	0.726
Fibrous crescent	-0.134	0.201	-0.256	0.014
Tubular atrophy	-0.132	0.209	-0.250	0.016

Further combined analysis of the two antibodies showed 36 cases of anti-C1qA08+/anti-mCRP a.a.35-47+ antibodies (double-positive antibody) and 26 cases of anti-C1qA08-/anti-mCRP a.a.35-47- (double-negative antibodies). The associations between double-positive and double-negative antibodies and clinicopathologic features were shown in **Tables 4**; **5**. Serum concentrations of C3 and C4 in patients with double positive antibodies were significantly lower than that in the double negative group ((0.52(0.25-1.19) g/L vs. 0.39(0.15-0.98) g/L, *P*=0.004) and (0.12(0.03-0.22) g/L vs. 0.05(0.02-0.18) g/L, *P<*0.001), respectively), And the double positive antibodies in patients were negatively associated with fibrous crescent, tubular atrophy and IgG deposition (r=-0.210, *P*=0.017, r=-0.248, *P*=0.022, and r=-0.365, *P*=0.004, respectively).

**Table 4:** Comparisons of clinical manifestations of patients with and without double positive antibodies.

	Double negative antibodies	Double positive antibodies	*p* value
SLEDAI scores	18 ± 6	19 ± 6	0.463
Acute kidney injuries	19.2%	25.0%	0.592
Hemoglobin(g/L)	42.3%	55.6%	0.303
Hematuria	80.8%	83.3%	1.000
Leukocyturia	60.0%	63.9%	0.758
Proteinuria(g/d)	4.5(0.5-15.4)	3.1(0.2-22.5)	0.158
Serum creatinine(µmol/L)	74.6(36.7-273.7)	83.5(34.7-792.0)	0.185
Serum C3(g/L)	0.52(0.25-1.19)	0.39(0.15-0.98)	0.004
Serum C4(g/L)	0.12(0.03-0.22)	0.05(0.02-0.18)	<0.001
Anti-dsDNA antibody	73.1%	91.4%	0.118

**Table 5:** Comparisons of pathological manifestations of patients with and without double positive antibodies.

Renal pathology score	Double positive antibodies	
	*r* value	*P* value
Activity indices score	0.126	0.400
Cell/cell fiber crescent	0.008	0.953
Neutrophil infiltration and/or nuclear fragmentation	0.017	0.896
Interstitial inflammatory	0.143	0.267
Chronicity indices score	0.192	0.196
Global sclerosis	0.071	0.581
Fibrous crescent	-0.210	0.017
Tubular atrophy	-0.248	0.022
IgG deposition	-0.365	0.004
IgA deposition	-0.102	0.429
IgM deposition	-0.160	0.213
C3c deposition	0.129	0.316

**Figure 2:**
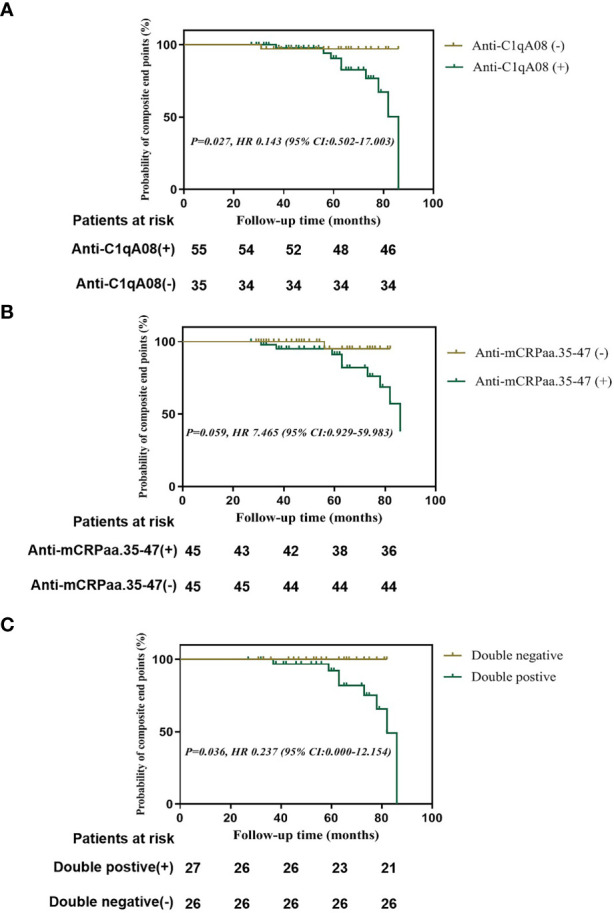
Renal outcomes of patients with anti-A08 antibodies and anti-mCRP a.a.35-47 antibodies in LN **(A)** Comparison of renal outcomes between patients with and without anti-C1qA08 antibodies **(B)** Comparison of renal outcomes between patients with and without anti-mCRP a.a.35-47 antibodies. **(C)** Comparison of renal outcomes between patients with double-positive antibodies of anti-mCRP a.a.35-47 antibodies and anti-C1qA08 antibodies and those without.

Finally, we used Kaplan-Meier analysis to compare the renal survival between patients with and without anti-C1qA08 or anti-mCRP a.a.35-47 antibodies. We found that patients with anti-C1qA08 antibodies had significantly worse renal prognosis than those without (*P*=0.027, HR 0.143 (95% CI:0.502-17.003)) (**Figure 2A**); The survival rate of patients with anti-mCRP a.a.35-47 antibodies was worse than those without (*P*=0.059, HR 7.465 (95% CI:0.929-59.983)) (**Figure 2B**); Patients with double-positive antibodies had significantly worse renal prognosis than those with double-negative antibodies (*P*=0.036, HR 0.237 (95% CI:0.000-12.154)) (**Figure 2C**).

### Binding of mCRP to C1q by enzyme-linked immunosorbent assay and surface plasmon resonance

**Figure 3:**
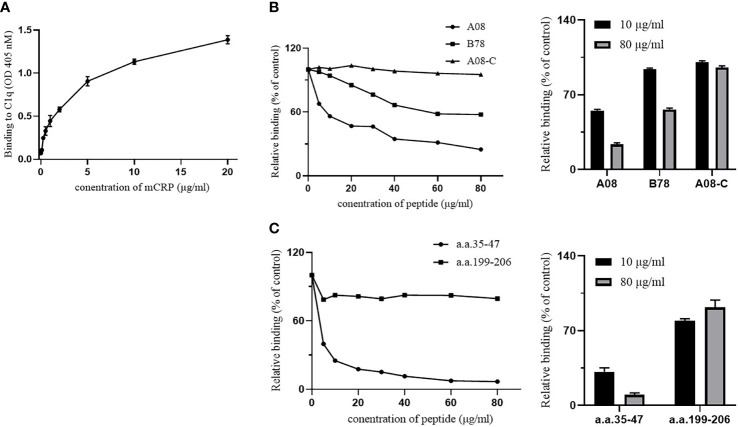
The binding epitopes of mCRP and C1q by ELISA. **(A)** C1q was immobilized on the ELISA plate, and then different concentrations of mCRP were added. The binding activity of mCRP to C1q was measured using mCRP-specific antibody 3H12. As the concentration of mCRP increased, the absorbance increased and was dose-dependent. **(B)** Fix the urea-denatured mCRP on the microplate, and different concentrations of peptides A08, B78, and A08-C were added to the plate. The inhibition of binding of C1q to mCRP was inhibited by 80% when 80 μg/ml of A08 was added. **(C)** Fix C1q on the microplate, then different concentrations of peptides a.a.35-47 and a.a.199-206 were added to the plate. We observed that a.a.35-47 significantly inhibited the binding of mCRP to C1q, and it inhibited by 90% when added to 80 μg/ml of a.a.35-47.

The ELISA method was performed to detect the binding of mCRP and C1q and the binding of their key epitopes, respectively. Firstly, the binding of C1q and mCRP was dose-dependent (**Figure 3A**).

To further clarify the key epitope of the combination of the two proteins, it was known that the key linear epitope of the C1q antibody is A08, and the anti-C1qA08 antibody was related to the poor prognosis of LN patients. It may be used as a non-invasive “biology marker” that can predict the long-term prognosis of patients with LN. mCRP was coated on a 96-well microtiter plate, then C1q and different concentrations of related peptides A08, B78, and A08-C mixture were added (**Figure. 3B**). As the concentration of related peptides increased, A08 significantly inhibited the binding of C1q and mCRP. When it reached 80 µg/ml, it inhibited 80% of the binding. B78 also has a certain effect but was relatively weak, while A08-C had almost no influence. The key epitope of binding between mCRP and C1q was A08.

To further clarify the key epitopes on mCRP, C1q was coated on a 96-well plate, and then mCRP and different concentrations of related peptides as a.a.35-47 mixture and a.a.199-206 mixture were added (**Figure 3C**). As the peptide concentration increased, a.a.35-47 significantly inhibited the binding of mCRP and C1q. When the concentration of a.a.35-47 up to 80 µg/ml, the inhibition rate up to 90%, while a.a.199-206 had no effect. a.a.35-47 was the key epitope for mCRP and C1q binding.

**Figure 4:**
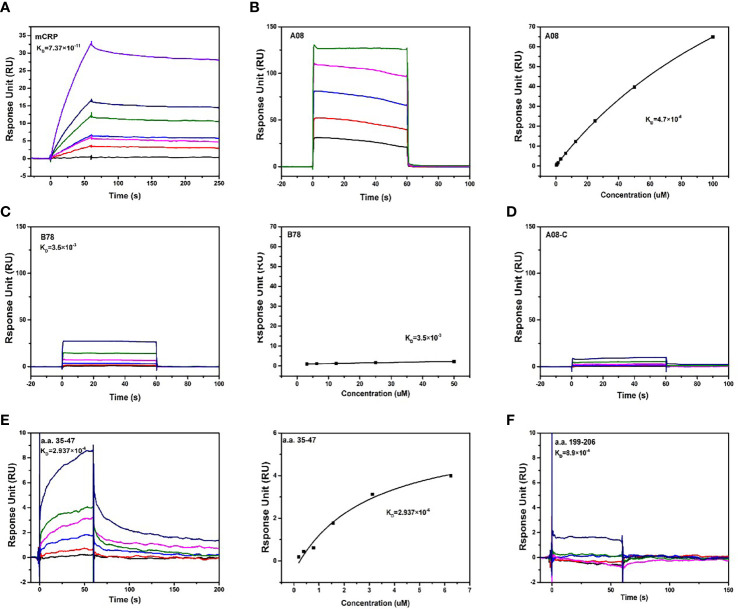
The binding epitopes of mCRP and C1q by SPR method. **(A)** Fix C1q on the CM5 chip and add different concentrations of mCRP, KD=0.0737 nM. **(B)** To clarify the key epitopes of binding, CRP was coupled to the CM5 chip, 200 s of the urea-deforming agent was injected, and CRP was depolymerized into mCRP. Recombined peptides of different concentrations A08, KD=4.7 μM. The curve was dose-dependent. **(C)** Based on B, change A08 to B78, KD=3.5 mM, the binding was very weak, and the steady-state curve showed that the response value changes a little as the concentration increases. **(D)** Similarly, different concentrations of A08-C were injected, KD=0. mCRP was hardly combined with A08-C. **(E)** C1q was immobilized on a CM5 chip, and different concentrations of a.a.35-47 were injected, which was characterized by slow binding and fast dissociation. The steady-state curve was used to determine the binding to dose-dependent, KD=2.937 μM. **(F)** C1q was fixed on the CM5 chip and different concentrations of a.a.199-206, KD=0.89 mM was injected. Therefore, C1q mainly bound to a.a.35-47 of mCRP, and mCRP mainly bound to the A08 epitope of C1q.

We further used optical surface plasmon resonance (SPR) to verify the key epitopes of C1q-related peptides to which mCRP directly binded. Firstly, human C1q was coupled to the CM5 chip, and purified mCRP with a concentration gradient was injected, the combination of the C1q and mCRP was dose-dependent. (**Figure 4A**).

To further clarify the binding epitope, mCRP was immobilized on the CM5 chip. However, mCRP was precipitated in an acetate buffer. We then coated the pentameric CRP on the chip, injected a certain amount of high-concentration deforming agent urea, and the CRP was finally converted into mCRP. And then injected different concentrations of peptides A08, B78, and A08-C. mCRP was mainly combined with A08 by comparing the KD value (**Figures 4B-D**). Then C1q was coated on the chip, and different concentrations of peptides a.a.35-47 and a.a.199-206 were injected. The comparison of KD values showed that C1q was mainly bound to a.a.35-47 of mCRP (KD=2.937×10^-6^) (**Figures 4E, F**).

### Anti-C1qA08 antibody inhibited the binding of C1q to mCRP

**Figure 5:**
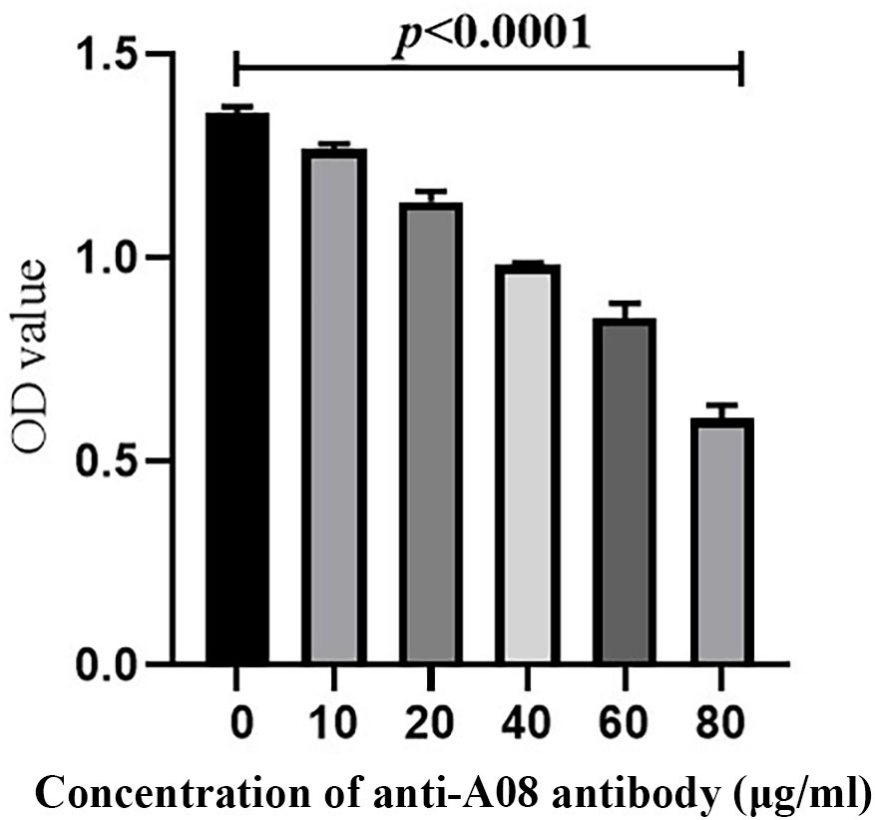
The anti-C1qA08 antibody inhibited the binding of C1q to mCRP. The addition of different concentrations of C1qA08 antibody, as shown by the graph could inhibit the binding of mCRP to C1q, and when 80 μg/ml anti-C1qA08 antibody was added, the inhibition rate exceeded 50%.

C1q A08 mAb (17-9) can bind to eight or 10 amino acids of the C-terminus of A08. C1q was first coated on ELISA plates. mCRP was co-incubated with the anti-C1qA08 antibody and competed with C1q for binding. The binding of mCRP to C1q was significantly inhibited as the concentration of A08 antibody increased (**Figure 5**). When the anti-C1qA08 antibody was added at 80 µg/ml, the inhibition rate exceeded 50%. Thus, the anti-C1qA08 antibody could inhibit the binding of mCRP to C1q and also demonstrated that A08 was the two key binding epitopes.

### C3 deposition

**Figure 6:**
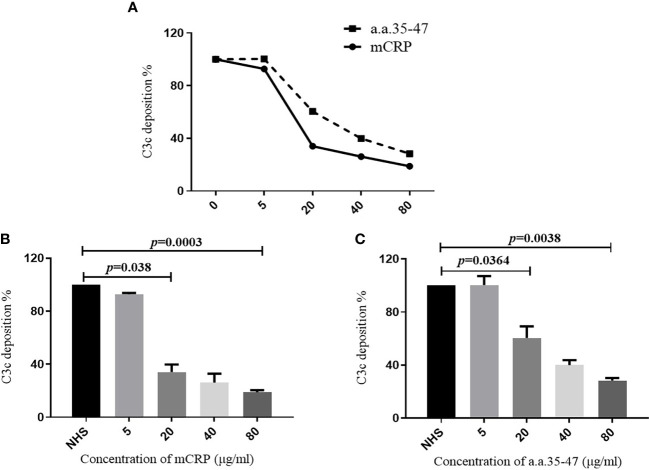
Detection of C3c deposition. To assess whether mCRP and a.a.35-47 can affect the ability of C1q to activate the classical pathway of complement. **(A)** Adding different concentrations of mCRP, a.a.35-47, C3c deposition decreases as the concentration increases. **(B)** Different concentrations of mCRP were added. As the concentration of C3c decreased, the complement was inhibited. When the concentration of mCRP reached 20 μg/ml, it could be inhibited to 30%. **(C)** When different concentrations of a.a.35-47 peptide were added, C3c deposition decreased with increasing concentration, and complement was inhibited. When the concentration of a.a.35-47 reached 80 μg/ml, it could be inhibited to 30%. Therefore, mCRP and a.a.35-47 in the liquid phase could inhibit complement activation.

Our results showed that a.a.35-47 was the key sequence on mCRP which mediated the binding of mCRP and C1q, while A08 was the key sequence on C1q which mediated the binding of C1q and mCRP. In LN, the classical pathway could be activated. We speculated that mCRP and a.a.35-47 might be involved in the activation of the classical complement pathway. *In vitro*, α3(IV)NC1 immune complex was immobilized onto microtiter wells, and 1% serum from healthy volunteers was added into wells. The complement can be activated by C1q binding to immune complexes and thus lead to the production of C3c. Different concentrations of mCRP were added to the serum in the subsequent experiments, and with the increase of the concentration of mCRP, the deposition of C3c decreased (**Figure 6A**). Similarly, different concentrations of a.a.35-47 were added to the serum, with the increase of concentration of a.a.35-47, the deposition of C3c decreased (**Figure 6A**). The above results demonstrated that mCRP could inhibit complement activation, and a.a.35-47 could also inhibit the complement activation. The inhibition rate could reach over 60% when 20 µg/ml mCRP was added to the serum. In addition, it elevated to more than 75% when 80 µg/ml mCRP was added to the serum (**Figure 6B**). The inhibition rate could reach over 40% when 20 µg/ml a.a.35-47 were added to the serum, while 40 µg/ml a.a.35-47 could inhibit 50% of activation (**Figure 6C**).

## Discussion

LN is one of the most serious complications of SLE, with over 50% of SLE patients developing LN in China (35, 36). The complement system is widely considered to be a ‘double-edged sword’ in LN (37, 38), with complement activation promoting pathogen clearance, but also causing tissue damage due to immune complex deposition. C1q is an important component of the classical pathway to complement and can bind to ligands such as mCRP, IgG, and fibronectin, et al. Both autoantibodies against C1qA08 and mCRP a.a.35-47 showed influence on prognosis. But the interaction between C1q and mCRP is still under discussion.

In our study, anti-C1qA08 antibodies and mCRP a.a.35-47 antibodies were prevalent in patients with LN, which was in accordance with previous studies. Moreover, the double-positive group showed more severe hypocomplementemia, which indicated that complement activation might exist in the double-positive group. We further found that levels of anti-C1qA08 and anti-mCRP a.a.35-47 antibodies correlated with the score of IgG deposition, fibrous crescents, and tubular atrophy, which suggested that these two autoantibodies were associated with renal pathological lesions and suggested the pathogenic role of these two autoantibodies (39). More importantly, patients with both anti-C1qA08 antibody and anti-mCRP a.a.35-47 antibodies had a worse prognosis. The clinicopathological analysis suggested that these two antibodies might not only be biomarkers but also of importance in the pathogenesis of LN.

Thus, we tested the combination of the C1q and mCRP and the key epitope of the binding activity. The combination of C1q and mCRP was proved by ELISA and SPR. Whether it was coated by C1q and then added mCRP, or coated mCRP and added C1q, the binding could be detected in a concentration-dependent manner. At the same time, the SPR method directly detected the combination of the two proteins, and the results showed that it was in the form of fast binding and slow dissociation, and the dissociation constant was very small, which proved that the binding force was super strong. The binding force of the two proteins was so strong that it was difficult to dissociate, so we speculated that mCRP might be involved in the pathogenesis of LN through binding to C1q. Both C1q and mCRP were macromolecular proteins, so it was necessary to further clarify the binding site of the two proteins. We first studied the important epitopes of C1q and mCRP reported in the literature, and then we used the competitive binding assays to verify the bound epitopes. The results suggested that A08 was an important epitope for the binding of C1q to mCRP, and a.a.35-47 was an important epitope for the binding of mCRP to C1q. After that, SPR was used tentatively to combine the two peptides. However, it was failed because the peptide could be fixed. We then used the monoclonal antibody of A08 to further inhibit the binding of C1q and mCRP, which turned out that A08 was the main binding site of C1q and mCRP.

We used an A08-specific antibody 17-9 mAb inhibition assay to demonstrate that A08 was a key epitope for C1q binding to mCRP. The results showed that 17-9 mAb could significantly inhibit the binding of both, so A08 was the key epitope. The complement system exerted an important role in the clearance of immune complexes in different tissues, and it was an important pathogenesis involved in LN that the dysfunction for the clearance of immune complexes and apoptotic cells. The result from C3 deposition showed that mCRP and a.a.35-47 could inhibit the activation of complement classical pathway through binding to C1q, which might interfere with the clearance of immune complex or apoptotic cells afterwards.

The main limitation of the current study was that there was no antibody to a.a.35-47and therefore no direct inhibition assays for binding of a.a.35-47 and C1q were performed. More work needs to be done to clarify the associations of mCRP and C1q in the pathogenesis of LN.

In conclusion, a combination of anti-C1qA08 and anti-mCRP a.a.35-47 antibodies could better predict the prognosis of LN. The key linear epitopes of the combination of C1q and mCRP were a.a.35-47 and C1qA08. A08 was an important epitope for the classical pathway complement activation and a.a.35-47 could inhibit this process.

## Data availability statement

The original contributions presented in the study are included in the article/**Supplementary Material**. Further inquiries can be directed to the corresponding author.

## Ethics statement

The studies involving human participants were reviewed and approved by Renal Division, Peking University First Hospital, Beijing, China. The patients/participants provided their written informed consent to participate in this study.

## Author contributions

Writing—original draft: XL. Review and editing: YT and SJ. All authors have read and agreed to the published version of the manuscript. All authors contributed to the article and approved the submitted version.

## Supplementary material

Supplementary Figure 1 The correlation analysis of levels of anti-C1qA08 antibodies and anti-mCRP a.a.35-47 antibodies.
