# Supplementary figures and images for: Combination of anti-C1qA08 and anti-mCRP a.a.35-47 antibodies is associated with renal prognosis of patients with lupus nephritis
Source: Front Immunol. 2023 Apr 17;14:1181561. doi: 10.3389/fimmu.2023.1181561 (PMC10150958; doi:10.3389/fimmu.2023.1181561)

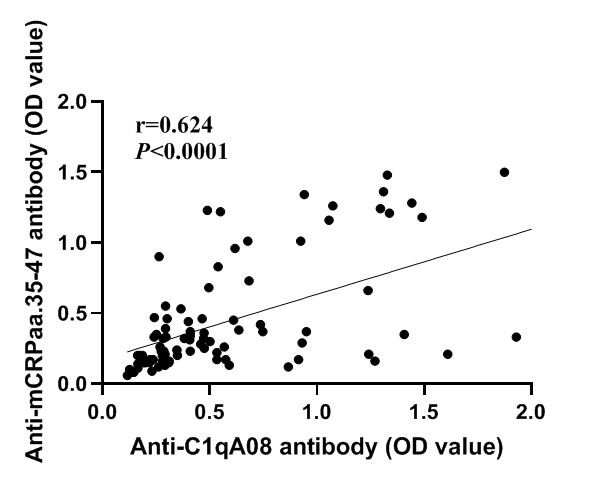

Supplement: Supplementary Figure 1 — The correlation analysis of levels of anti-C1qA08 antibodies and anti-mCRP a.a.35-47 antibodies. [file Image_1.jpeg]
